# Marvels of Surgery Performed by Russians

**Published:** 1916-01

**Authors:** 


### Marvels of Surgery Performed by Russians.

Marvels in surgery are almost everyday occurrences in Russian hospitals, according to Dr. John Mann, of Virginia, who has come to Japan after nine months' experience in the American Red Cross Hospital at Kiev. Dr. Mann served with a staff of American nurses who are soon returning to the United States on account of lack of Red Cross funds. He held the rank of lieutenant colonel in the Russian army.

"In many instances," said Dr. Mann to the correspondent, "it was really a case of making faces while you wait. Some Russian soldiers came to us with their faces literally blown off. They were supplied with artificial jaws and were sent away looking almost entirely new. They were the best classes of operation I have ever seen."

The American hospital at Kiev was practically devoted to surgery. There were eight American doctors and one Russian, and also American and Russian nurses. At first there was accommodations for 400 beds, but this soon was increased to 700. The patients as they came in were treated to a bath and clean clothes and then were allotted to the different wards. Some said they had not had their clothes off or had taken a bath for six months.

These were the men who had been all through the campaign in the Carpathian Mountains last winter. Most of them were suffering from frost bite.

"There was absolutely no kind of gunshot wound which we did not have," went on the doctor, "and it may be mentioned that the Russians had splendid facilities for sending on their wounded. The railway station itself had been converted into a receiving hospital. All patients arriving there were given a dressing and then distributed to the different hospitals. A first-aid dressing, of course, had previously been given on the field. Occasionally, however, there was no time for any dressing at the station. This occurred, for instance, when one night 1000 patients arrived and had to be distributed to the hospitals at once."

Almost every public building in Kiev, the physician continued, was used as a hospital, and there was even one of 100 beds in the catacombs of the city. Operations were performed on every part of the human anatomy. He found the spirit of the Russian people excellent and that of the women wonderful. Everybody was confident of the success of the Russian arms and ready to sacrifice everything to that end.

The people got on very well without drinking intoxicants. The law against the sale of alcoholic beverages was strictly enforced. One Russian dealer was sent to Siberia for selling a bottle of champagne. When the day's work was done there was opportunity for recreation and rest in the theaters, where good vaudeville shows were given.

"It was delightful," remarked Dr. Mann, "to find one night a troupe of dancers from the United States."

Dr. Mann, who is a nephew of former Governor Mann of Virginia, will be in charge of St. Luke's Hospital in Tokio during the absence of Dr. R. B. Teusler, who has gone to the United States to complete plans for the establishment here of St. Luke's International Hospital. He has served in the New York City Hospital and in St. Luke's Hospital, Norfolk, Va.—*Correspondence of the Associated Press.*

